# Brain structure, working memory and response inhibition in childhood leukemia survivors

**DOI:** 10.1002/brb3.621

**Published:** 2016-12-29

**Authors:** Ellen van der Plas, Russell J. Schachar, Johann Hitzler, Jennifer Crosbie, Sharon L. Guger, Brenda J. Spiegler, Shinya Ito, Brian J. Nieman

**Affiliations:** ^1^ Physiology and Experimental MedicineThe Hospital for Sick Children Research InstituteTorontoONCanada; ^2^ Psychiatry ResearchThe Hospital for Sick ChildrenTorontoONCanada; ^3^ Department of PsychiatryFaculty of MedicineThe University of TorontoTorontoONCanada; ^4^ Department of PediatricsFaculty of MedicineThe University of TorontoTorontoONCanada; ^5^ Department of Haematology/OncologyThe Hospital for Sick ChildrenTorontoONCanada; ^6^ Department of PsychologyThe Hospital for Sick ChildrenTorontoONCanada; ^7^ Clinical Pharmacology and ToxicologyThe Hospital for Sick ChildrenTorontoONCanada; ^8^ Pharmacology and PharmacyFaculty of MedicineThe University of TorontoTorontoONCanada; ^9^ Mouse Imaging Centre (MICe)The Hospital for Sick ChildrenTorontoONCanada; ^10^ Ontario Institute for Cancer ResearchTorontoONCanada; ^11^ Department of Medical BiophysicsThe University of TorontoTorontoONCanada

**Keywords:** acute lymphoblastic leukemia, cognitive late effects, magnetic resonance imaging

## Abstract

**Introduction:**

Survival rates for children with acute lymphoblastic leukemia (ALL) approach 95%. At the same time, there is growing concern that chemotherapy causes alterations in brain development and cognitive abilities. We performed MRI measurements of white and gray matter volume to explore how variation in brain structure may be related to cognitive abilities in ALL survivors and healthy controls.

**Methods:**

The sample included 24 male ALL survivors who had completed contemporary treatment 3–11 years prior, and 21 age‐ and sex‐matched controls. Participants were between 8 and 18 years old. Working memory and motor response inhibition were measured with the N‐Back and Stop Signal Tasks (SST), respectively. Participants underwent 3T structural MRI to assess white and gray matter volumes overall, lobe‐wise, and in cortical and atlas‐identified subcortical structures. Mental health was assessed with the Child Behavioral Checklist.

**Results:**

ALL survivors performed more poorly on measures of working memory and response inhibition than controls. Frontal and parietal white matter, temporal and occipital gray matter volume, and volumes of subcortical white and gray matter structures were significantly reduced in ALL survivors compared with controls. Significant structure‐function correlations were observed between working memory performance and volume of the amygdala, thalamus, striatum, and corpus callosum. Response inhibition was correlated with frontal white matter volume. No differences were found in psychopathology.

**Conclusions:**

Compared with controls, a reduction in volume across brain regions and tissue types, was detectable in ALL survivors years after completion of therapy. These structural alterations were correlated with neurocognitive performance, particularly in working memory. Confirming these observations in a larger, more representative sample of the population is necessary. Additionally, establishing the time course of these changes—and the treatment, genetic, and environmental factors that influence them—may provide opportunities to identify at‐risk patients, inform the design of treatment modifications, and minimize adverse cognitive outcomes.

## 1 Introduction

Acute lymphoblastic leukemia (ALL) accounts for 30% of childhood cancers and most commonly affects children 2 to 5 years of age (Inaba, Greaves, & Mullighan, 2013; National Cancer Institute 2015). Prior to the 1960s, ALL was uniformly lethal (Haut, Altman, Cartwright, & Wintrobe, 1955; Mangalik, Boggs, Wintrobe, & Cartwright, 1966), but due to advances in medicine, 5‐year relative survival rate now approaches 95% (SEER 2011). Contemporary treatment of ALL consists of approximately 2 years of of multi‐agent chemotherapy (Chabner & Roberts, 2005; Pui & Evans, 2013), extended a further 6–12 months in males due to increased risk of relapse (Bleyer et al., 1991). The primary routes of administration are oral and intravenous, with some agents, most notably methotrexate (MTX), also delivered directly into the cerebrospinal fluid space by lumbar puncture to prevent relapse arising from spread of leukemia to the membranes surrounding the brain and spinal cord (Inaba et al., 2013). Chemotherapy treatment effectively cures ALL; however, there is a growing concern about persistent adverse outcomes, referred to as ‘late effects’. These late effects include cardiomyopathy, avascular necrosis of the joints, secondary cancers, and neurocognitive and behavioral challenges (Goldsby et al., 2010; Haddy, Mosher, & Reaman, 2009; Landier, Armenian, & Bhatia, 2015; Mariotto et al., 2009; Schultz et al., 2007a). Neurocognitive and behavioral late effects include weaknesses in working memory, visual‐spatial reasoning, motor coordination, processing speed, and attention (Campbell et al., 2009; Iyer, Balsamo, Bracken, & Kadan‐lottick, 2015; Jacola et al., 2016; Peterson et al., 2008). Similar processes are known to be disrupted in individuals with traumatic brain injury or stroke (Lipszyc et al., 2014; Ornstein et al., 2013; Sinopoli, Schachar, & Dennis, 2011) and, like individuals with brain injury, childhood cancer survivors need long‐term follow‐up care to identify and treat late effects (Blaauwbroek, Groenier, Kamps, Meyboom‐de Jong, & Postma, 2007). This burden persists into adulthood when symptoms may be similar to the cognitive decline observed in aging (Ahles, 2012; Edelstein et al., 2011).

Children with ALL require treatment at a time when the brain is undergoing major developmental changes (Dean et al., 2014; Giedd et al., 2010; O'Muircheartaigh et al., 2014). Chemotherapy may disrupt or impair these processes and thereby lead to neurocognitive deficits. It is critical to understand which aspects of brain development are altered and how these are related to neurocognitive late effects before it will be possible to remediate or prevent these adverse outcomes in ALL survivors. Several studies have established MRI findings indicative of pathology or altered brain structure. Transient lesions in the white matter (leukoencephalopathy) due to chemotherapy are well‐documented in ALL survivors (Bhojwani et al., 2014; Reddick, Glass, Johnson, Laningham, & Pui, 2009; Reddick et al., 2005); however, these lesions do not show a clear relationship with functional abilities (Bhojwani et al., 2014; Pääkkö et al., 2000) and are therefore unlikely to account for the full range of deficits in ALL survivors. Reductions in total white matter volume and increased fractional anisotropy have been reported in adult survivors of childhood ALL (Aukema et al., 2009; Edelmann et al., 2014; Reddick et al., 2014), with some evidence of associations between white matter morphology and measures of attention and intelligence (Edelmann et al., 2014; Reddick et al., 2014) and motor processing speed (Aukema et al., 2009).

Several gaps remain, however. Firstly, available studies often evaluate outcomes in adult survivors of childhood leukemia (Blaauwbroek et al., 2007; Edelmann et al., 2014; Kanellopoulos et al., 2015; Tamnes et al., 2015; Zeller et al., 2013). ALL treatment has changed dramatically since these older survivors were diagnosed (Pui & Evans, 2013; Simone, 2006): due to concerns of significant neuropsychological effects (Janzen & Spiegler, 2008; Spiegler et al., 2006), cranial radiation therapy has almost entirely been replaced by chemotherapy‐only protocols (Pui & Evans, 2013; Pui et al., 2009). Secondly, ALL survivors in late childhood or adolescence may be faced with unique challenges associated with ongoing brain maturation processes (Erus et al., 2015). Thirdly, while global changes in tissue volumes have been reported (Krull et al., 2016; Reddick et al., 2014; Tamnes et al., 2015), detailed characterization of region‐specific changes associated with chemotherapy treatment remains scant (Genschaft et al., 2013). For instance, regional differences in developmental time course (e.g., protracted development of the frontal lobes) (Erus et al., 2015; Zatorre, Fields, & Johansen‐Berg, 2012) are likely to alter regional sensitivity to chemotherapy and its long‐term impact. Finally, there is little understanding of the potential relationships between regional brain deficits and functional abilities in ALL survivors.

The goal of this study was to characterize brain structure and neurocognitive performance on experimental tasks assessing working memory and inhibitory control, comparing results between childhood ALL survivors treated with contemporary chemotherapy protocols, and age‐ and sex‐matched control subjects. We hypothesized that ALL survivors would exhibit lower regional volume in gray matter and white matter relative to controls, and that these would correlate with neurocognitive performance.

## 2 Materials and Methods

### 2.1 Participants

We were interested in alterations in brain development associated with cancer treatment in late childhood through adolescence. ALL is more common in males (National Cancer Institute 2015) and males are given a more extended chemotherapy treatment due to increased risk for relapse (Brecher et al., 1986; Tiedemann, Chessells, & Sandland, 1982; Wofford et al., 1992). To limit treatment‐related variability and inherent sex differences in brain development (De Bellis et al., 2001; Gur et al., 1999; Lange, Giedd, Castellanos, Vaituzis, & Rapoport, 1997; Kang, Herron, & Woods, 2011), while maximizing eligible participants, we restricted this study to males only.

ALL survivors were recruited at the Hospital for Sick Children (Toronto, Canada) as part of a multidisciplinary study on late effects of ALL treatment. Survivors were eligible to participate if they were male, of European ancestry, between 8 and 18 years old, at least 2 years off treatment, and were treated with chemotherapy only (i.e., no radiation therapy). Participants were also part of a genetic study, and ancestry was factored into recruitment. Survivors with Down syndrome or individuals with medical or mental health conditions who were unable to complete the assessments were excluded from the study. Of the 110 individuals who consented to participate in the larger study, 35 met our eligibility criteria (~32% of entire sample). Seven ALL survivors declined to participate in this study (20% of the eligible sample), and two withdrew from the study (6%).

Healthy comparison subjects were recruited through advertisement in the hospital. Control subjects were matched to ALL survivors on age, sex, and ancestry. Additionally, controls were eligible if they did not have a diagnosed learning disability or psychiatric disorder at the time of assessment.

Both ancestry and mental health were screened for at the time of recruitment for both controls and survivors and were verified on the day of testing with a questionnaire. To establish ancestry during initial recruitment, participants were asked: “Are *both* grandparents on *both* sides White/European?” To assess participants’ psychiatric history, we asked: “Do you currently have a diagnosis of a psychiatric disorder such as depression, anxiety, ADHD or drug abuse?” and “Have you ever experienced a brain injury (such as concussion) that lead to hospitalization?” If participants answered yes to the latter question, we followed up by asking “Have you experienced symptoms in the last 3 years?” They were considered eligible if they indicated they were free of symptoms. Because disorders such as ADHD are common among survivors, we did not exclude survivors who reported mental health or psychiatric diagnoses, provided they were able to complete MRI scanning without sedation.

On the day of testing, participants and/or their parents filled out a questionnaire to verify eligibility information obtained at recruitment. To establish the ancestry, parents/participants were required to select from ancestral categories for biological paternal and maternal grandparents. Responses included: (1) I have no info; (2) White/European; (3) Black/African; (4) Hispanic; (5) Asian; (6) Arab. Likewise, parents and/or participants filled out a questionnaire about medical history, which asked whether they *currently* had a diagnosis of ADHD or any other psychiatric condition and whether medication was required for these conditions. The questionnaire also repeated inquiries about head trauma, concussion, surgery, mental health, and/or learning disabilities.

### 2.2 Ethics and informed consent

The procedures performed as part of the study were in accordance with the 1975 Helsinki declaration and its later amendments. The study was approved by the Hospital for Sick Children's Research Ethics Board. Informed consent/assent was obtained from participants and their parents/guardians. The consenting procedure was performed by trained, experienced staff.

### 2.3 Patient and treatment

ALL patients were stratified at diagnosis to receive standard‐ or high‐risk ALL therapy based on prognostic factors that include the patient's age, initial white blood cell count, blast cytogenetics, and early response to treatment (Borowitz et al., 2006; Inaba et al., 2013; Mattano et al., 2014; Pui & Evans, 2013; Schultz et al., 2007b). In our group, survivors had received one of four standard‐risk or one of two high‐risk ALL treatment protocols (Table 1) (Borowitz et al., 2006; Hinds et al., 2007; Schultz et al., 2007b; Termuhlen et al., 2012, 2013). We collected data on age at diagnosis and calculated time from diagnosis.

**Table 1:** ALL treatment protocols and risk stratification of ALL survivors in the sample

Risk stratification	Standard risk				High risk	
Protocol ID	9904	AALL0331	9905	9605	A5971	9906
*n*	8	8	3	2	1	1
Age at diagnosis	2.1–5.4	2.6–8.4	3.2–4.2	3.0–4.5	3.1	2.9

9904/9905: Borowitz et al. (2006), Hinds et al. (2007).

AALL0331: Maloney et al. (2013), Mattano et al. (2014), Mitchell et al. (2016).

9605: Montour‐Proulx et al. (2005).

A5971: Termuhlen et al. (2012, 2013).

9906: Borowitz et al. (2006).

### 2.4 Measures

#### 2.4.1 Child behavior checklist

General psychopathology was assessed with the Child Behavioral Checklist (CBCL) (Achenbach & Ruffle, 2000) to corroborate self‐reported mental health status. The CBCL is a parent questionnaire that addresses internalizing and externalizing symptoms during the preceding three months. Parents rate 120 statements on a three‐point scale: not true (0), somewhat true or sometimes true (1), or very true or often true (2). Age‐adjusted T‐scores for each sub‐scale were calculated. We included the six DSM‐oriented scales in our analyses to feature a relatively broad range of potential issues: affective problems; anxiety problems; somatic problems; attention deficit/hyperactivity problems; oppositional defiant problems; and conduct problems. T‐scores between 50 and 65 are considered in the normal range, while T‐scores greater or equal to 65 are indicative potentially of clinical difficulties (Achenbach & Ruffle, 2000).

#### 2.4.2 Youth self report

We asked participants age 11 and older to complete the Youth Self Report (YSR). This questionnaire is nearly identical to the parent‐completed CBCL, but is completed by youth themselves (Achenbach, 1991).

#### 2.4.3 N‐back

**Figure 1:**
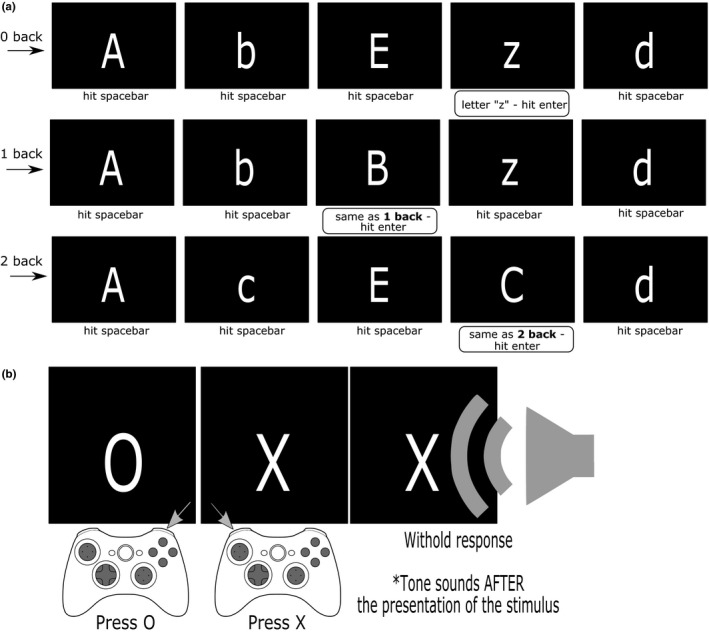
Schematic representations of the N‐Back and Stop Task. In the N‐back task (A), the participant hits “enter” if the displayed letter meets criterion, or “space” otherwise. For the 0‐back, “enter” is required whenever a particular letter (“z”) is shown. For the 1‐back and 2‐back, “enter” is required when the letter matches one shown 1‐ or 2‐times previously

Participants were required to respond to a series of letters (Figure 1a). Participants completed the 0‐back, 1‐back, and 2‐back condition. Conditions were comprised of three blocks with 40 trials that were 500ms long. A practice block preceded commencement of trials for each condition. Participants responded to lower and upper‐case letters on the screen by pressing either the spacebar key or the Enter key. For the 0‐back condition, participants pressed the Enter key whenever the letter “z” appeared on the screen, otherwise they pressed the spacebar. In the 1‐back condition, participants pressed the Enter key when the letter presented on the screen was the same as the letter presented on the immediately previous trial. For the 2‐back condition, participants pressed ‘Enter’ whenever the letter on the screen was the same as the one observed 2 trials previously. On all other trials they pressed spacebar (Figure 1a).

Performance was evaluated by target accuracy, which was defined as the percentage of trials on which participants responded correctly to the stimulus by pressing the Enter key. To ensure only those who performed above chance were considered, participants were excluded from the analysis if they did not achieve at least 30% target accuracy. The validity screen was applied before calculating age‐corrected scores.

#### 2.4.4 Stop signal task

The Stop Signal Task (SST) includes two components: The Go task and the Stop task (Figure 1b). In the Go task component, participants are required to discriminate between an X and an O that are presented one at a time for 1,000 ms followed by a 500 ms fixate. There are five blocks (including one practice block) with 24 trials each. Participants were instructed to respond as quickly and accurately as possible. The stop signal, an auditory tone, is presented on a random subset of the trials (25%) (X or O) (Figure 1b). Participants are instructed to cancel their response when the stop signal is presented. The delay between presentation of the go stimulus and the stop signal (stop signal delay) is initially set at 250 ms and is dynamically adjusted depending on whether or not the participant was able to stop (signal‐inhibit) or failed to stop (signal‐respond) on a particular trial (Verbruggen & Logan, 2009). If a response was stopped (signal‐inhibit), the stop signal delay was increased by 50 ms, making it more difficult to stop the next response. If they failed to stop (signal‐respond), the delay was decreased by 50 ms making it easier to stop on the next stop‐signal trial. Using this tracking algorithm, the stop signal delay converges on the delay at which individuals successfully stop their response 50% of the time, referred to as the mean delay reaction time (MDT).

Stop signal reaction time (SSRT) refers to the time interval between the start of the stop process (presentation of stop signal) and the point at which the stop process finishes (Verbruggen & Logan, 2009). Finishing of the stop process is estimated from the distribution of the go reaction time and the observed probability of responding given a stop signal. For participants in our sample, this probability was set to 0.50. SSRT can be estimated by subtracting MDT from the observed mean reaction time (MRT). We also examined variability in reaction time by looking at the standard deviation of the mean reaction time (SDRT) (Lipszyc & Schachar, 2010). Individuals with fewer than 20% or greater than 80% successfully inhibited stop trials were excluded because this resulted in either too few correct stop trials or failed stop trials to calculate stable SSRTs. Note that higher SSRT scores represent greater deficits.

#### 2.4.5 Magnetic resonance imaging

Brain structure was assessed with a 3T MRI scanner (Siemens Medical Solutions). Total scan sessions did not exceed 30 minutes, and included T1‐weighted imaging, diffusion tensor imaging and magnetization transfer imaging. T1‐weighted sequences were acquired with an MPRAGE sequence with parameters: flip angle 9º, TI = 0.9s, TR = 2.3s, TE = 3 ms, 7.1 ms spacing, 256 × 240 mm field‐of‐view, 192 1 mm slices, 1 mm isotropic resolution, GRAPPA 2, and 5m3s acquisition time. The DTI scan was run with an echo‐planar readout with the following parameters: TR = 9s, TE=90 ms, 0.7 ms echo spacing, 244 × 244 mm field‐of‐view, 70 2 mm slices, 2 mm isotropic resolution, GRAPPA 2, *b* = 1000 s/mm^2^, with 30 directions and 6 *b* = 0 scans (total scan time 5m53s). A gradient‐echo field map was also acquired (echo times 5.19 and 7.65 ms, total scan time 1m44s). Magnetization transfer (MT) images were collected using a gaussian MT pulse shape (10 ms, 1,200 Hz offset, total flip angle 500°) with a gradient‐echo readout: flip angle 10º, TR = 51 ms, TE = 3.8 ms, 192 × 192 mm field‐of‐view, 1.5 mm isotropic resolution, GRAPPA 2, and total scan time 3m55s (for each, the reference and MT scan, ~8 m for both).

#### 2.4.6 Image analysis

MR images were processed to assess total and regional morphology. Processing was first performed with the CIVET pipeline (v1.1.12). Scans were normalized to the ICBM152 template (Collins, Neelin, Peters, & Evans, 1994) and then corrected for intensity nonuniformity (Sled, Zijdenbos, & Evans, 1998). After skull stripping, the images were classified into gray matter, white matter, and cerebrospinal fluid, using FSL FAST (version 5.0). From the classified volumes, we measured total gray and white matter volumes, and then evaluated volumes for each of the frontal, parietal, occipital and temporal lobes. The cortex was identified from the gray–white matter and gray matter cerebrospinal fluid boundaries, using deformable models (Kim et al., 2005) and surface‐based nonlinear registration (Lyttelton, Boucher, Robbins, & Evans, 2007). Measures of cortical thickness, area and volume were then evaluated lobe‐wise. Subcortical white and gray matter anatomy were evaluated using structures drawn from four atlases: the Johns Hopkins white matter atlas (Mori et al., 2008) (based on fractional anisotropy from the DTI data); the thalamus, globus pallidus, and striatum atlas from the Montreal Neurological Institute and McGill University (Chakravarty, Bertrand, Hodge, Sadikot, & Louis Collins, 2006); the hippocampal atlas from the Toronto Centre for Addiction and Mental Health (author now at the Douglas Mental Health University Institute, Montreal) (Winterburn et al., 2013); and the amygdala atlas described in Treadway et al. and Entis et al. (Entis, Doerga, Barrett, & Dickerson, 2012; Treadway et al., 2015). In all, we tested volumes of a total of 39 automatically segmented, subcortical atlas structures. In each case, atlases were used to reconstruct individual images based on multiple automatically generated templates (Chakravarty et al., 2013; Pipitone et al., 2014). In this algorithm, multiple atlas templates are generated by nonlinear registration of an expertly segmented atlas to a number of subject images (in our case, 21 were selected). After registration of each subject's image to the automatically generated templates, a voxel voting procedure was used to generate segmentations for each individual. Summation of the number of segmented voxels was then used to determine total structure volume.

For visualization of subcortical changes more locally, we also performed deformation‐based morphometry and computed volume change between each subject and an unbiased consensus average image. For this purpose, we used the iterative model building approach provided in the Pydpiper toolkit (Friedel, van Eede, Jon Pipitone, Chakravarty, & Lerch, 2014). In this algorithm, six‐parameter linear registration is used to orient subject images according to a standard template and then pairwise 12‐parameter registration of all images is used to generate an initial registration of all images, which are averaged to produce a model estimate. Iterative nonlinear registration of the model to all individuals at progressively finer scales is used to generate a refined model. Although the human cortex is too variable to register in this fashion, subcortical regions can be assessed this way. Consequently, this analysis was restricted to a subcortical region‐of‐interest and served as a qualitative confirmation of atlas‐based analyses. For visualization, regions of significant volume change were overlaid on the average image using a color map to indicate percent volume change.

### 2.5 Statistical analysis

To compare controls and ALL survivors on the CBCL and YSR, we calculated the proportion of individuals with T ≥ 65 for both groups and performed tests of equal proportions. For the N‐Back, we used a generalized linear model to fit the target accuracy scores (% correct), using age and group as explanatory variables, and specifying a quasi‐binomial distribution. We ran linear regression models with group and age as explanatory variables on the Stop Signal Task, reporting significance of coefficients after verifying normality of model residuals. The neuro‐anatomical results were modeled likewise.

We calculated age‐corrected scores for visualization of the results and correlation analysis between performance and anatomical measurements. Adjusted scores for the N‐Back were computed based on the age coefficient generated from the generalized linear model fit in the transformed (linear) space. After adjustment according to each subject's age relative to the reference (14 years, the approximate mean of the sample), scores were transformed back to produce an adjusted accuracy score that ranged from 0% to 100%. Response inhibition scores were corrected based on the linear regression model and the difference between each subject's age and the reference age. A similar procedure was used for normalizing neuroanatomical volume measurements. The relationships between brain structure and neurocognitive performance was explored with Pearson correlation coefficients on age‐corrected values. We report significance using *p*‐values or corrected *p*‐values (*q*‐values), computed with the false discovery rate (FDR) (Benjamini & Hochberg, 1995) to account for multiple comparisons.

## 3 Results

### 3.1 Sample characteristics

**Figure 2:**
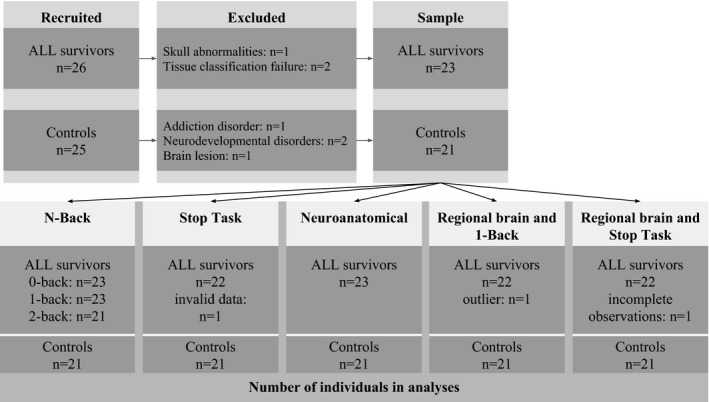
Consort diagram. The diagram indicates the number of participants at each step of the study, and which were included in analyses. Where participants or data were excluded, the reason is listed

We recruited 26 ALL survivors (74% of eligible sample) and 25 comparison subjects in our study. Three survivors and four control subjects were omitted from all analyses (Figure 2): one survivor presented with skull and brain abnormalities; image quality was poor and/or automated tissue classification failed in two survivors; three control subjects reported neurodevelopmental and/or addiction disorders on the day of testing (and thus did not meet our inclusion criteria); and one control subject was found to have a tumor. The final data sample for analysis included 23 survivors and 21 controls. Mean age was not significantly different between groups. ALL survivors averaged 14.4 years old (*SD* = 2.2) and controls averaged 13.9 years old (*SD* = 2.9).

Mean age at diagnosis in ALL survivors was 4.4 years old (*SD* = 1.8). On average, 10.0 years had passed since diagnosis (*SD* = 2.5). Of the 23 ALL survivors in our sample, 21 had received standard‐risk and 2 high‐risk treatment protocols (Table 1). Two survivors in the high‐risk category had received high‐dose MTX (Borowitz et al., 2006; Termuhlen et al., 2012, 2013), whereas those on standard‐risk protocols did not.

### 3.2 CBCL

**Figure 3:**
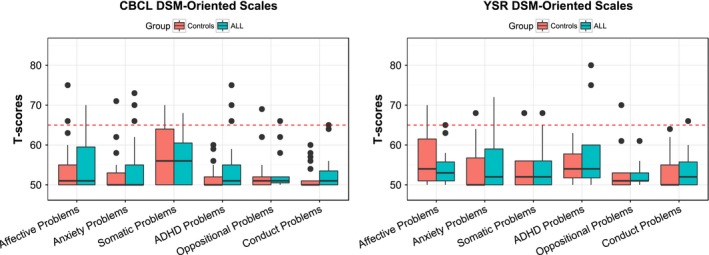
CBCL and YSR results. The distribution of CBCL (left) and YSR (right) T scores on DSM‐oriented scales in controls (pink) and ALL survivors (blue) are shown in box‐and‐whisker plots. The dotted red lines represent the cutoff at which scores are considered clinically relevant. Several individuals in both groups score above the “normal” range, but there is no significant difference between groups

Median T‐scores for DSM‐oriented scales in ALL survivors and controls were well within the normal range (Figure 3): 30 out of 44 (68%) participants had T scores in the normal range (< 65) on each subscale. The proportion of individuals in the impaired range (T ≥ 65) on each of the scales for controls and survivors, respectively, were as follows: affective problems (12.5% and 15.0%); anxiety (6.3% and 15.0%); somatic problems (18.9% and 5.0%); ADHD problems (0% and 10%); oppositional problems (6.3% and 0%); conduct problems (0% and 0%). None of the CBCL categories were significantly different between groups.

### 3.3 YSR

These analyses included 16 controls and 20 ALL survivors who were at least 11 years old. The proportion of individuals in the impaired range (T ≥ 65) on each of the YSR scales for controls and survivors, respectively, were as follows: affective problems (18.8% and 5.0%); anxiety (6.3% and 5.0%); somatic problems (12.5% and 20.0%); ADHD problems (0% and 10%); oppositional problems (6.3% and 0%); conduct problems (0% and 5.0%). None of the YSR categories was significantly different between groups.

### 3.4 N‐back performance

**Figure 4:**
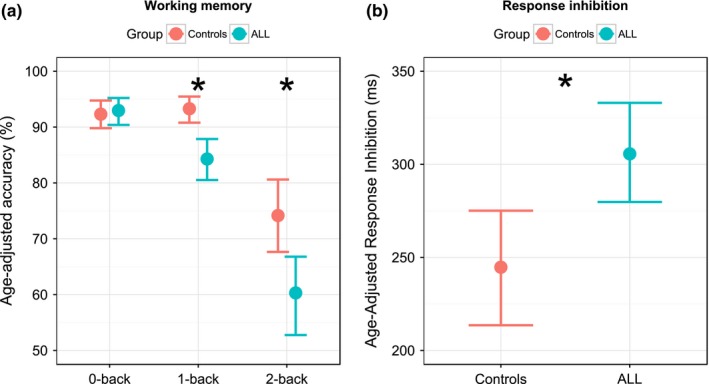
N‐Back and Stop Task results. Performance on the N‐Back (Panel a) and Stop Task (Panel b) in controls (pink) and ALL survivors (blue). The graphs show means with 95% confidence limits. In Panel a, the N‐Back condition is plotted on the *x*‐axis and target accuracy on the *y*‐axis. ALL survivors (blue) made significantly more errors than controls (pink) in the 1‐back and 2‐back conditions (*p* = .0009 and .01 respectively). In Panel b, the groups are plotted on the *x*‐axis and response inhibition is plotted on the *y*‐axis. For response inhibition, lower scores represent better performance. ALL survivors (blue) were significantly slower on this this task compared with controls (pink) (*p* < .01). The asterisks mark significant differences between groups

Working memory was deficient in ALL compared with controls in the 1‐back and 2‐back condition. The generalized linear model with 0‐back target accuracy as the outcome variable and age and group (survivor vs. control) as predictor variables showed a significant effect of age (*t*_(41)_ = 2.8, *p* = .009), but not for group (*t*_(41)_ **= 0.76, *p* = .45) (Figure 4a). In the 1‐back condition, accuracy scores were 9.6% lower in survivors (mean = 84.3, *SD *= 9.1) than controls (mean = 93.3, *SD *= 5.5), which was highly significant between groups (*t*_(41)_ = −3.6, *p* = .0009). The age coefficient was also significant (*t*_(41)_= 3.0, *p* = .004). On the 2‐back condition, two ALL survivors scored <30% and were omitted from the analysis. Subsequently, survivors scored on average of 18.7% lower (mean* *= 60.3; *SD *= 17.5) than controls (mean* *= 74.2; *SD *= 15.3), with the model showing significant age (*t*_(39)_ **= 3.7, *p* = .0007) and group (*t*_(39)_ **= −2.6, *p* = .01) coefficients (Figure 4a).

Age at diagnosis and years since diagnosis were not significantly associated with age‐corrected target accuracy scores on the N‐Back.

### 3.5 Stop task performance

One ALL survivor did not meet the validity criteria for the Stop Signal Task (total *n* = 43, 21 controls and 22 survivors [Figure 2]). ALL survivors had longer SSRT (ms) (mean=307.3; *SD *= 72.7) than did controls (mean* *= 244.0; *SD *= 71.2), representing a 26.0% increase in stopping reaction time (Figure 4b). The linear model indicated a significant effect of both group (*t*_(40)_ = 2.8, *p* < .01) and age (−13 ms/yr, *t*_(40)_ **= −2.9, *p* < .01).

ALL survivors also tended to be more variable in response time than did controls (SDRT). The groups did not differ on MRT. Neither age at diagnosis nor years since diagnosis were predictive of age‐adjusted SSRT.

### 3.6 Anatomical measurements from MRI

**Figure 5:**
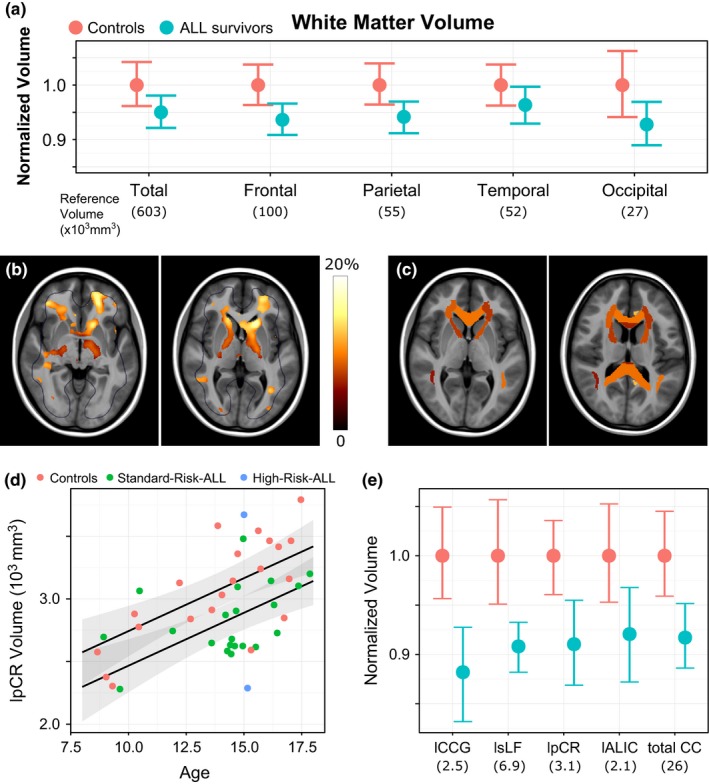
White matter in ALL survivors and controls. Panel a: Lobe‐wise, normalized white matter volume in ALL survivors (blue) and controls (pink). Regions are listed on the *x*‐axis, along with the reference volume for controls in parentheses (used for normalization). Normalized volume is on the *y*‐axis. Means and 95% confidence intervals of the means are shown. Frontal and parietal white matter were significantly reduced in ALL survivors compared with controls (*q* < 0.1). Panel b: Deformation‐based morphometry results. The heatmaps represent change in volume between ALL survivors and controls, ranging from 0 to 20% difference (shown in regions with *q* < 0.1). Throughout subcortical regions, ALL survivors had significantly lower volume than did controls. Panel c: Atlas‐based analyses of subcortical differences between ALL survivors and controls were used to quantify volume in individual structures. Volume differences between ALL survivors and controls were significant for the corpus collasum (genu and rostrum) and the internal capsule (*q* < 0.1). Panel d: For each atlas structure, the relationship between age and volume were modeled. Shown is the left posterior corona radiata volume (lpCR) across ALL survivors (green = standard‐risk treatment; blue = high‐risk treatment) and controls (pink), showing on average a lower volume in ALL survivors. Panel e: Summary statistics (means and 95% confidence intervals) after normalization for age show decreased volume of subcortical white matter structures in ALL survivors (blue) relative to controls (pink). A complete list of comparisons is provided in Table 2. lpCR = Left posterior corona radiata; lCCG = left cingulum along cingulate gyrus; lsLF = left superior longitudinal fasciculus; lpCR = Left posterior corona radiata; lALIC = left anterior limb of the internal capsule; Total CC = total corpus callosum volume

We evaluated volume of brain regions using MRI, observing a general trend for decreased volumes in ALL survivors as compared to controls. Lobe‐wise testing of white matter volume revealed that ALL survivors had smaller frontal white matter volume bilaterally, decreased by 6.5% on the left and and 6.4% right side (*t*_(41)_ = −2.3, *q* = 0.09 and *t*_(41)_ = −2.6, *q* = 0.09). ALL survivors also had 5.8% smaller right parietal white matter volume than controls (*t*_(41)_ = −2.3, *q* = 0.09) (Figure 5a). Subcortical white matter differences were present in 11 of the 29 white matter structures tested, with 5% to 12% volume decreases (Figure 5b–e). These included the following: the genu (−9.4%), body (−6.3%) and splenium (−9.4%) of the corpus callosum; the left and right anterior corona radiata (−7.0 and −8.8%); the right superior corona radiata (−6.6%); the left and right posterior corona radiata (−9.0 and −8.8%); the left anterior limb of the internal capsule (−7.9%); left cingulum (along the cingulate gyrus) (−11.8%); and the left superior longitudinal fasciculus (−9.2%). A complete listing of computed results and statistical comparisons between survivors and controls is provided in Table 2.

**Table 2:** List of 39 automatically segmented, subcortical atlas structures and the difference between ALL survivors and controls in these structures

Structure	Subregion	% Volume Change(ALL vs. controls)			*t*‐value			*q* value		
Subcortical white matter		L	M	R	L	M	R	L	M	R
Corpus callosum^a^	Genu		−9.4			−2.6			0.07	
	Body		−6.3			−2.6			0.07	
	Splenium		−9.4			−2.3			0.09	
Corona radiata^a^	Anterior	−7.0	—	−8.8	−2.2	—	−2.5	0.09	—	0.07
	Superior	−4.5	—	−6.6	−1.7	—	−2.2	ns	—	0.09
	Posterior	−9.0	—	−8.8	−2.9	—	−2.7	0.07	—	0.07
Internal capsule^a^	Anterior limb	−7.9	—	−7.2	−2.2	—	−2.1	0.09	—	ns
	Posterior limb	−6.5	—	−6.2	−1.8	—	−1.7	ns	—	ns
	Retrolenticular part	−3.3	—	−4.8	−1.1	—	−1.3	ns	—	ns
External capsule^a^		−3.2	—	−3.9	−1.2	—	−1.6	ns	—	ns
Cingulum^a^	Cingulate gyrus part	−11.8	—	−5.9	−3.4	—	−1.6	0.06	—	ns
	Hippocampal part	−5.3	—	−1.2	−1.3	—	−0.3	ns	—	ns
Cerebral peduncle^a^		−3.2	—	−2.9	−0.7	—	−0.6	ns	—	ns
Posterior thalamic radiation (& optic radiation)^a^		−2.2	—	−3.8	−0.6	—	−1.0	ns	—	ns
Longitudinal fasciculus^a^	Superior	−9.2	—	−5.5	−3.1	—	−2.0	0.06	—	ns
Sagittal stratum (incl. Inferior longitudinal fasciculus and inferior occipital fasciculus)		−1.9	—	−4.7	−0.4	—	−1.2	ns	—	ns
Subcortical gray matter										
Amygdala^b^		−5.4	—	−7.4	−1.9	—	−2.7	ns	—	0.07
Hippocampus^c^		−3.5	—	−3.3	−1.3	—	−1.3	ns	—	ns
Thalamus^d^		−4.5	—	−4.8	−1.9	—	−2.2	ns	—	0.09
Globus pallidus^d^		−6.9	—	−5.0	−2.9	—	−1.9	0.07	—	ns
Striatum^d^		−6.3	—	−5.6	−2.2	—	−1.9	0.09	—	ns

Acronyms: M, midline; L, Left; R, Right.

a Atlas: Johns Hopkins white matter atlas (Mori et al., 2008).

b Atlas: Treadway et al. and Entis et al. (Entis et al., 2012; Treadway et al., 2015).

c Atlas: Toronto Centre for Addiction and Mental Health (Winterburn et al., 2013).

d Atlas: Montreal Neurological Institute and McGill University (Chakravarty et al., 2006).

**Figure 6:**
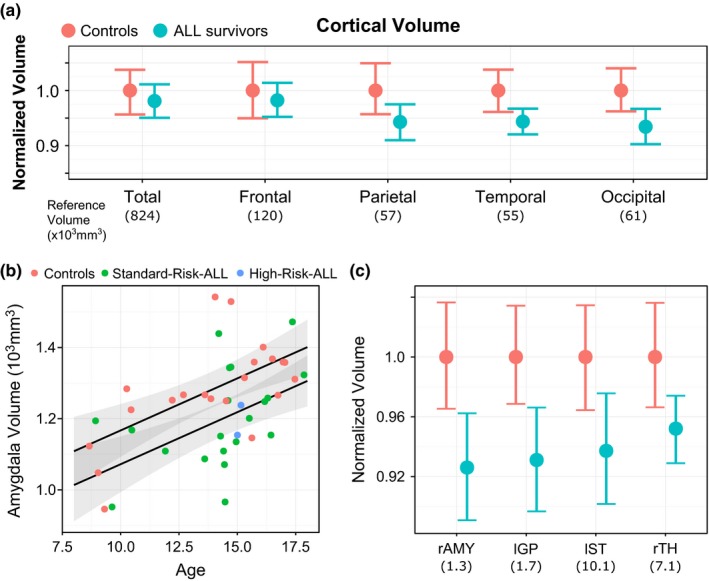
Gray matter volume in ALL survivors and controls. Panel a: Lobe‐wise, normalized cortical volume in controls (pink) and ALL survivors. Regions are listed on the *x*‐axis, along with reference volume for controls in parentheses. Normalized volume is on the *y*‐axis. Means and 95% confidence intervals are shown. Temporal and occipital cortical volume were significantly reduced in ALL survivors compared with controls (*q* = 0.09). Panel b: Relationship between age and right amygdala volume across ALL survivors (green = standard‐risk treatment; blue = high‐risk treatment) and controls (pink), showing an average volume decrease in ALL survivors. Panel e: Summary statistics (means and 95% confidence intervals) for normalized volume of subcortical gray matter structures in ALL survivors (blue) and controls (pink). A complete list of comparisons is provided in Table 2. rAMY **=  right amygdala; lGP **= left globus pallidus; lST **= left striatum; rTH **= right thalamus

In addition to white matter volume changes, ALL survivors exhibited smaller cortical gray matter volume than controls in the temporal and occipital lobes (Figure 6a). On the right side, volumes were smaller by 5.6% and 6.6%, respectively (*t*_(41)_ = −2.3 and −2.4, *q* = 0.09). On the left side, only the occipital lobe was significantly smaller (−6.4%, *t*_(41)_ **= −2.2, *q* = 0.09). Measures of cortical thickness and surface area did not differ between survivors and controls. Significant volume decreases were also observed in subcortical gray matter structures (Figure 6b,c). Of the 10 gray matter structures tested, 4 were significantly different after correction for multiple comparisons (across all 39 structures). Survivors exhibited smaller volumes in the amygdala (right, −7.4%), the thalamus (right, −4.8%), the globus pallidus (left, −6.9%), and the striatum (left, −6.3%). Full results and statistics are listed in Table 2, and show that survivors always exhibited smaller volumes (though not all of volume decreases reached the threshold for significance). Age at diagnosis, years since treatment and treatment intensity were not correlated with age‐adjusted total brain volume, white matter or gray matter. We also did not observe significant changes in MT or DTI measurements.

### 3.7 Regional brain volume and cognition

We analyzed correlations between performance‐based measures and anatomical outcomes across ALL survivors and controls. Since our data showed that controls and ALL survivors were best distinguished on the 1‐back condition (owing to higher variability on the 2‐back condition), we used the age‐adjusted 1‐back accuracy scores as a proxy for working memory abilities in these analyses. One ALL survivor (age 17) was identified as having impaired 1‐back performance (*z* = −3.2) after accounting for age, and was excluded as an outlier (Grubbs test, *p* = .01) (Grubbs, 1950).

**Figure 7:**
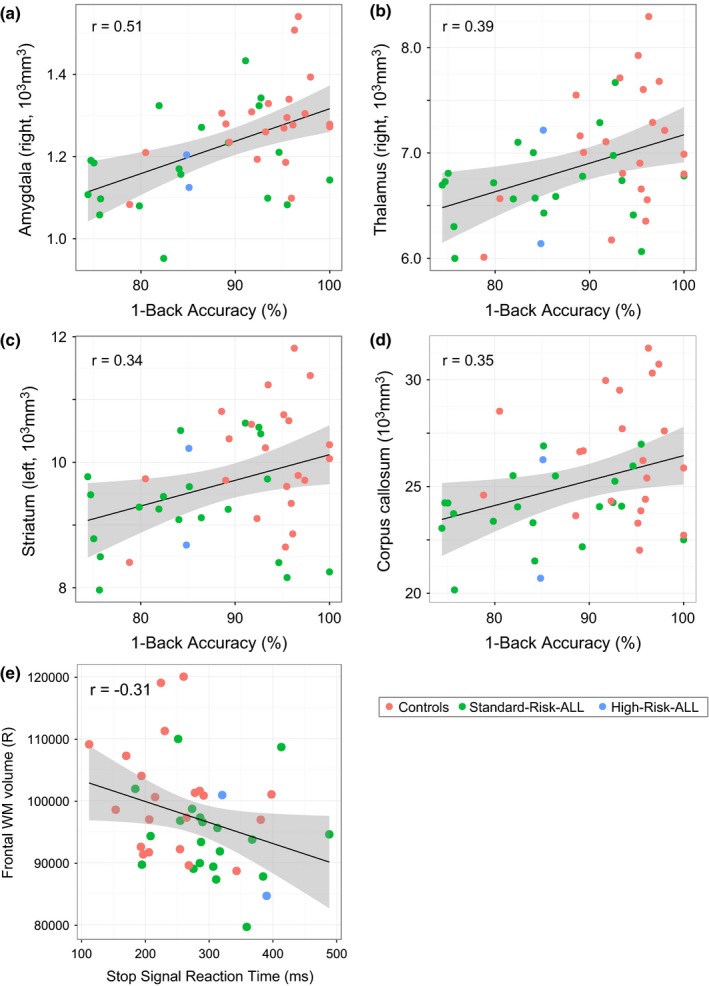
Regional brain structure and cognitive abilities. Panels a–d show correlations between subcortical volume measurements (right amygdala, right thalamus, left striatum and corpus collasum, respectively), and target accuracy on the 1‐back across ALL survivors (green = standard‐risk treatment; blue = high‐risk treatment) and controls (pink) (all significant correlations at *q* < 0.1). In panel e, the right frontal white matter volume is shown as correlated with response inhibition (*p* = .04, uncorrected). All measures are age‐corrected

Performance on the 1‐back task was correlated with right amygdala volume (*r* = .51, *t*_(40)_ **= 3.7, *q* = 0.03) (Figure 7a), right and left thalamus volume (*r* = .39 and 0.35, *t*_(40)_ **= 2.7 and 2.4, *q* = 0.09 and 0.1) (Figure 7b), left striatum volume (*r* = .34, *t*_(40)_ **= 2.3, *q* = 0.1) (Figure 7c), and total corpus callosum volume (*r* = .35, *t*_(40)_ **= 2.4, *p* = .02) (Figure 7d). For the latter, tests showed significance independently for both the genu and the body of the corpus callosum (*r* = .37, *t*_(40)_ **= 2.6, *q* = 0.09), but not the splenium (*q* = 0.26). Correlation with the 1‐back task was also observed in the right and left anterior corona radiata (both *r* = .38, *t*_(40)_ **= 2.7, *q* = 0.09), the right hippocampal portion of the cingulum (*r* = .34, *t*_(40)_ **= 2.3, *q* = 0.1), and the right superior longitudinal fasciculus (*r* = .35, *t*_(40)_ **= 2.4, *q* = 0.1). Age‐corrected SSRT showed some association with right frontal white matter volume (*r* = −.31, *t*_(41)_ **= −2.1, *p* = .04, uncorrected) (Figure 7e).

## 4 Discussion

In this study, we characterized regional‐ and tissue‐specific neuroanatomical changes after treatment for childhood ALL at a level of detail not previously explored. We also showed how neuroanatomical alterations relate to changes in neurocognitive processes. Our results are consistent with the idea that altered brain development may drive late effects in childhood ALL survivors.

The results indicate that both neurocognitive and anatomical deficits were evident in childhood and adolescent ALL survivors even after nearly a decade had passed since ALL diagnosis. Survivors exhibited difficulties in working memory and response inhibition, and had lower regional gray and white matter volume compared with typically developing controls. Correlation between working memory and volume was significant for the amygdala, thalamus, striatum, and corpus callosum. These regions have been implicated in performance on the N‐Back previously (Dahlin, Neely, Larsson, Bäckman, & Nyberg, 2008; Kubat‐Silman, Dagenbach, & Absher, 2002; Schaefer et al., 2006; Takeuchi et al., 2010; Zou, Hong, Wang, Gao, & Yang, 2011). Response inhibition was correlated with frontal white matter volume, consistent with previous observations in a lesion study of traumatic brain injury (Lipszyc et al., 2014). Our results did not show strong evidence among survivors for distinct “affected” and “not affected” groups, but rather suggested the distribution of ALL survivors was globally shifted relative to that of the controls. Further observations will be necessary to determine if subgroups of survivors have different outcomes. Our results do indicate that exposure to chemotherapy treatment results in brain development changes years after completion of treatment, with potential ramifications for quality of life in ALL survivors.

It is crucial to identify the underlying cellular and molecular mechanisms that drive the structural changes observed in neuroimaging studies so that lasting neurocognitive impairments in ALL survivors can be addressed. The higher prevalence of volume differences in white matter may indicate increased toxicity there. Indeed, treatment‐induced damage to oligodendrocytes has been proposed as a potential mechanism of neuroanatomical changes in ALL survivors (Reddick et al., 2014). Oligodendrocytes have a high metabolic rate and must produce large amounts of membranes and protein, particularly during myelination (Bradl & Lassmann, 2010). These characteristics may make these cells especially sensitive to chemotherapy‐induced disruptions of normal cell function. ALL patients are treated with a combination of ~10 different chemotherapy agents through several phases lasting 2–3 years (Inaba et al., 2013; Pui, Robison, & Look, 2008; Pui et al., 2009). All of these agents could, either individually or in combination, affect (regional) brain development. One commonly suspected chemotherapy agent is MTX, which targets folate synthesis (Graham et al., 1992; Pak, Chan, & Mattson, 2003). Folate is a crucial constituent of one‐carbon metabolism (OCM) and is important for biosynthesis of purines and thymidylate, and remethylation of homocysteine to methionine (Fox & Stover, 2008; Li, Vijayanathan, Gulinello, & Cole, 2010). Since MTX is delivered through all phases of ALL treatment, folate metabolism may be altered for an extended period, possibly resulting in build‐up of homocysteine (Fox & Stover, 2008; Krull et al., 2016; Li et al., 2010; Steinfeld et al., 2009), and/or secondary metabolic alterations (van der Plas et al., 2015; Steinfeld et al., 2009; Strain et al., 2013). A recent study reported correlations between plasma MTX, cortical thickness in the dorsolateral prefrontal cortex and microstructure of the frontostriatal tract (Krull et al., 2016), suggesting that exposure to MTX is related to neuroanatomical changes. However, each of the other ALL chemotherapy agents may impact the brain and alter development.

Prior studies have reported links between performance on neurocognitive tasks and individual patient or treatment factors (Jansen et al., 2006; Krull et al., 2013; Reddick et al., 2014). In our sample, we found that age at diagnosis and years since diagnosis had no significant impact on cognitive abilities or neuroanatomical outcomes. However, the bulk (83%) of our survivors were diagnosed between 2 and 6 years of age, which corresponds to the age of peak ALL incidence (Inaba et al., 2013; National Cancer Institute 2015). A much larger, targeted cohort may be necessary to quantify the relative sensitivity of those diagnosed at an older age. Our cohort was not intended to distinguish between the impact of high‐ versus standard‐ or low‐risk treatment protocols. Future research should address if survivors who have received high‐risk protocols exhibit different neurocognitive patterns than survivors who received standard‐risk treatment.

Some caution in broadening the conclusions of our study is warranted. Our study included males of European ancestry only. We included as homogeneous a group of participants as possible to maximize our ability to detect differences between groups. The possible impact of sex, in particular, on late effects may be significant. Firstly, sex is known to modulate brain development (De Bellis et al., 2001; Gur et al., 1999; Lange et al., 1997; Kang et al., 2011) so that manifestation of late effects at any given age may depend on sex (Anderson & Kunin‐Batson, 2009; Buizer, de Sonneville, van den Heuvel‐Eibrink, & Veerman, 2005). Secondly, sex modulates disease‐ and treatment‐specific factors: incidence of ALL is higher in boys than in girls (Inaba et al., 2013; National Cancer Institute 2015; Pui & Evans, 2013; Pui et al., 1999; Sather, Miller, Nesbit, Heyn, & Hammond, 1981), and boys require longer treatment (~2.5 years) than girls (~2 years) due to sex‐specific risk factors for ALL relapse (Brecher et al., 1986; Tiedemann et al., 1982; Wofford et al., 1992). On the other hand, females may be at greater risk for cognitive late effects after cancer treatment (Buizer, de Sonneville, & Veerman, 2009; Hudson et al., 2003; Reddick et al., 2014; Waber, Tarbell, Kahn, Gelber, & Sallan, 1992). It is likely that the results shown here are not representative of outcomes in females. Similarly, ancestry, which is known to alter both pediatric ALL incidence and survival (Abrahão et al., 2015; Goggins & Lo, 2012; Linabery, Johnson, & Ross, 2012), is likely to also impact the risk and severity of late effects (Bhatia et al., 2002; Dores, Devesa, Curtis, Linet, & Morton, 2012; Lim, Bhatia, Robison, & Yang, 2014; Yang et al., 2010). Addressing the sex and ancestry dependence of late effects may have important clinical and biological implications (Buizer et al., 2005; Jansen et al., 2008; Peterson et al., 2008; von der Weid et al., 2003). Finally, we explored brain–cognition relationships using measures of working memory and inhibition only. ALL survivors may also demonstrate deficits in other domains, including visual‐motor coordination, processing speed and behavioral attention (Iyer et al., 2015). It will be beneficial to establish a comprehensive definition of neurocognitive and behavioral late effects in ALL survivors that highlights which measures provide greatest sensitivity/specificity to late effects, which to date remain only loosely defined.

This study complements and extends the existing literature by demonstrating regional and tissue‐specific volumetric changes in ALL survivors during late childhood and adolescence, a time when many late effects emerge. We conclude that even patients treated with standard‐risk, contemporary chemotherapy‐only treatment protocols are at risk of alterations in brain development, and deficits in working memory and inhibitory control. We have shown that structural changes are widespread and correlate with functional performance. As a large majority of childhood ALL patients will become survivors, late effects and their impact on quality of life are a crucial concern (Kunin‐Batson, Kadan‐Lottick, & Neglia, 2014; Mitchell et al., 2016), making the effort to identify targets for remediating cognitive, and behavioral deficits a pressing issue (Iyer et al., 2015; Kunin‐Batson et al., 2014; Olson & Sands, 2015). Animal models of cancer care will play an important role in systematically addressing the impact of chemotherapy on brain development and the impact of clinical variables (Gazdzinski et al., 2012; Nieman et al., 2015; de Guzman et al., 2015; Li et al., 2010). In a study on the adverse impact of cranial irradiation on brain structure, we showed that brain changes in radiation‐treated cancer survivors were very similar to those observed in radiation‐treated mice (Nieman et al., 2015), highlighting the potential of using mice as a representative model. Further research establishing the early time course of alterations in brain development, the principal causes, and sensitizing or protective factors is needed to design prevention and treatment strategies for ALL survivors.

## Conflict of interests

None declared.
